# OP29 Persistence Of Biologic And Targeted Synthetic Disease-Modifying Antirheumatic Drugs In The Treatment Of Rheumatoid Arthritis: A Cohort Study

**DOI:** 10.1017/S0266462325100871

**Published:** 2025-12-29

**Authors:** Natália Brandão, Gerusa Oliveira, Marina Garcia, Bárbara Alvernaz, Francisco Acurcio, Augusto Guerra, Juliana Álvares-Teodoro

## Abstract

**Introduction:**

Rheumatoid arthritis (RA) is a chronic inflammatory autoimmune disease affecting the joints. Biological disease-modifying antirheumatic drugs (bDMARDs) and targeted synthetic disease-modifying antirheumatic drugs (tsDMARDs) have expanded the therapeutic armamentarium for RA and significantly changed its management. However, there are still unmet needs for patients who do not respond to treatment. This cohort study aimed to evaluate the effectiveness of DMARDs in real-world settings.

**Methods:**

The study compared the effectiveness of Janus kinase inhibitors (iJAK; a type of tsDMARD) and anti-tumor necrosis factor (TNF) and non-anti-TNF drugs (bDMARDs) through treatment persistence in first- and second-line therapy. Access to these drugs requires an administrative approval process in the Brazilian public health system (BPHS). The cohort included patients with RA who requested their first drug between June 2018 and September 2022. The study was conducted in BPHS (Minas Gerais State) pharmacies and used drug dispensing records and clinical and sociodemographic data. Kaplan-Meier curves were used to compare treatment persistence at 18 months’ follow-up.

**Results:**

Among 763 patients, 85.1 percent were female and 33.0 percent white—mean age of 55.4 years and 6.5 years since initial diagnosis. Initially, 68.2 percent used anti-TNF drugs (adalimumab, etanercept, infliximab, certolizumab pegol, golimumab), 7.2 percent used non-anti-TNF drugs (abatacept, tocilizumab), and 24.6 percent used iJAK agents (tofacitinib, baricitinib, upadacitinib). The non-anti-TNF group had the lowest treatment persistence (p<0.05). Etanercept and baricitinib showed higher persistence. In the second line, 46.2 percent of patients used anti-TNF drugs, 23.9 percent used non-anti-TNF drugs, and 29.9 percent used iJAK agents (p>0.05). The baricitinib and tocilizumab groups showed higher persistence. In the second line, a general decrease in treatment persistence occurred.

**Conclusions:**

First- and second-line iJAK agents had the best persistence; there was no difference between drug groups in the second line. However, the rates of discontinuation and drug switching suggested treatment failure. The results suggested that patients respond less to second-line treatment. These results are useful for future reassessment decisions.

